# Is Coarse-to-Fine Strategy Sensitive to Normal Aging?

**DOI:** 10.1371/journal.pone.0038493

**Published:** 2012-06-04

**Authors:** Benoit Musel, Alan Chauvin, Nathalie Guyader, Sylvie Chokron, Carole Peyrin

**Affiliations:** 1 Laboratoire de Psychologie et NeuroCognition, CNRS – UMR 5105, Université Pierre Mendès France, Grenoble, France; 2 Grenoble-Image-Parole-Signal-Automatique (GIPSA-lab), CNRS – UMR 5216, Grenoble, France; 3 Unité Fonctionnelle Vision and Cognition, Fondation Ophtalmologique Rothschild, Paris, France; Baycrest Hospital, Canada

## Abstract

Theories on visual perception agree that visual recognition begins with global analysis and ends with detailed analysis. Different results from neurophysiological, computational, and behavioral studies all indicate that the totality of visual information is not immediately conveyed, but that information analysis follows a predominantly coarse-to-fine processing sequence (low spatial frequencies are extracted first, followed by high spatial frequencies). We tested whether such processing continues to occur in normally aging subjects. Young and aged participants performed a categorization task (indoor vs. outdoor scenes), using dynamic natural scene stimuli, in which they resorted to either a coarse-to-fine (CtF) sequence or a reverse fine-to-coarse sequence (FtC). The results show that young participants categorized CtF sequences more quickly than FtC sequences. However, sequence processing interacts with semantic category only for aged participants. The present data support the notion that CtF categorization is effective even in aged participants, but is constrained by the spatial features of the scenes, thus highlighting new perspectives in visual models.

## Introduction

A considerable number of studies on the visual system in humans and animals suggest that spatial frequencies are crucial in visual perception. The visual system does not process information in a Euclidean domain, but rather acts like a Fourier analyzer. Visual stimuli are processed in amplitude and phase spectra, with the amplitude spectrum decomposing the image in terms of spatial frequencies and orientations, and the phase spectrum describing the spatial relationships between the different spatial frequencies. Indeed, in primates, the primary visual cortex is largely dominated by complex cells which respond preferentially to different orientations and spatial frequencies [1], [2], [3], [4], [5]. On the basis of convergent data from the functional neuroanatomy of magnocellular and parvocellular visual pathways [6], neurophysiological recordings in primates [7], [8], psychophysical and neuroimaging results in humans [9], [10], [11], [12], [13], [14], [15], [16], [17], [18], [19], [20], and computational data [21], [22], influential theories of visual recognition postulate that recognition begins with the parallel extraction of visual features at different spatial frequencies (also called spatial scales), and follows a predominantly coarse-to-fine (CtF) processing sequence. Low spatial frequencies (LSF) in a visual input may be conveyed by the fast magnocellular visual pathway, and thus reach higher-order areas in the dorsal stream (parietal and frontal regions) and the ventral stream (inferotemporal regions) rapidly, allowing the initial perceptual parsing of visual inputs. This first coarse analysis might be then refined by higher spatial frequency information (HSF), which is conveyed more slowly to the cerebral cortex by the parvocellular visual pathway.

The first experimental evidence in support of a CtF processing sequence in human vision comes from psychophysical studies using hierarchical stimuli (global forms composed of several local elements; [23]). Usually, the global form is identified more quickly than local elements (global precedence effect), suggesting that global information is processed before local information. Based on the assumption that global information is preferentially conveyed by LSF, whereas local information is conveyed by HSF [24], [25], [26], the global-to-local processing sequence has been interpreted as reflecting a fundamental principle of the CtF processing sequence. Additional evidence of a CtF processing sequence was provided by psychophysical studies using more ecological stimuli, such as natural scenes and faces [11], [14], [18], [19]. Schyns and Oliva [18], for example, used hybrid stimuli made of two superimposed images of natural scenes, taken from different semantic categories and containing different spatial frequencies (e.g., a highway scene in LSF superimposed on a city scene in HSF). The perception of these hybrid scenes was dominated by LSF information when presentation time was very brief (30 ms), but by HSF information when presentation time was longer (150 ms), suggesting precedence of LSF over HSF in the visual processing time-course. Although the coarse-to-fine processing appears to be the predominant way of operating, the sequence of spatial scale information has been found to be relatively flexible, depending on the demands of the task [11], [18], [27]. In the Schyns and Oliva's study [18], a substantial proportion (29%) of hybrid sequences were in fact categorized in accordance with a fine-to-coarse (FtC), rather than a CtF time-course. Subsequent study of Oliva and Schyns [11] showed that the spatial scale preferentially processed in hybrid images can be constrained by a phase of prior sensitization which implicitly “primes” visual processing in favor of a particular scale (coarse or fine). After initial exposure to LSF information, the subsequent categorization of hybrid images was preferentially performed following LSF cues, whereas it was biased towards HSF information after priming by HSF. By using hybrid faces instead of scenes, Schyns and Oliva [19] showed that HSF information was preferentially used to determine whether a face was expressive or not, whereas LSF information was preferentially used to categorize emotion (e.g., happy, angry). The demands of a categorization task may, therefore, determine which range of spatial frequencies is extracted, and subsequently processed, from hybrid stimuli. Taken together, these studies suggest that all spatial frequencies were available at the beginning of the categorization, and that both types of sequence processing may coexist in the visual system.

Besides, few computational models have focused on the temporal processing of spatial frequencies. Most scene classification models are based on some low level visual features extracted from the whole spatial frequency distribution to provide natural scene category. For example, models used texture description [28] or the energy of the scene in different spatial frequencies and orientations [21], [22], [29], [30], [31], [32] to categorize natural scenes. Interestingly, results provided by Mermillod, Guyader and Chauvin [22] using connectionist simulation showed that categorization could be performed based on either LSF or HSF, depending on the semantic category of the natural scene used. The categorization of forest, mountain and indoor scenes was better when based on HSF information, whereas city and beach categories were better categorized from LSF information. The selection of spatial frequencies during the perception of natural scenes may, therefore, depend on interactions between the information actually needed for a given categorization task (top-down processes), and available perceptual information (bottom-up processes). This means that the sequence of spatial frequency analysis could be flexible, with FtC strategy being sometimes preferred to a CtF strategy, depending on task demands and on the perceptual properties of categories [33].

However, from a developmental perspective, data from the literature dealing with the sequence of spatial processing with aging remain unclear. Behavioural experiments have tested the aging of visual sequences indirectly using hierarchical stimuli. Some authors argued that in children, local processing emerged prior to the ability to process at global level [34], [35], [36]. A number of studies have shown that with age, the advantage usually observed for global processing tends to be reversed in favour of local processing [37], [38], [39]. For example, Lux et al. [37] reported that young adult participants had faster reaction times in the detection of global targets, while aged participants had faster reaction times in the detection of local targets. However, others studies have affirmed that global precedence was not reduced or reversed with increasing age [40], [41].

The first aim of the present experiment was to specify the sequence involved in spatial frequency processing during natural scene categorization. Using for the first time dynamic stimuli (thus permitting the sampling of more spatial frequencies) based on large natural scenes following CtF and FtC sequences, we hypothesized that participants would categorize CtF sequences more quickly than FtC sequences, as described in the vision models mentioned previously. The second aim was to investigate the sequence of spatial frequency processing during natural scene categorization in young and aged adult participants in order to specify visual models in normal aging.

## Materials and Methods

### Participants

Forty six right-handed participants were divided into two age groups: 23 young participants (10 males; 20 years±2; range 18 – 24); and 23 aged participants (10 males; 68 years±4; range 61 – 75) with normal or corrected-to-normal vision, were included in this experiment. Participants with neurological and ocular disorders (age-related macular degeneration, glaucoma and multiple sclerosis) were not included in the study. All participants gave their informed written consent before participating in the study, which was approved by the local ethics committee.

### Stimuli

**Figure 1:**
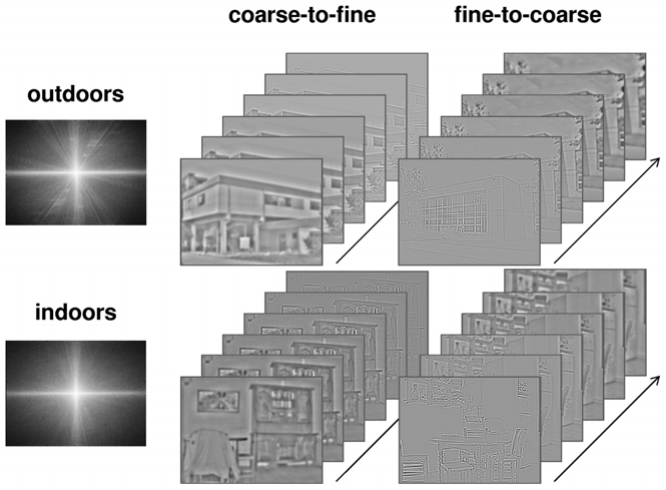
Example of six spatial frequency filtered images of scenes belonging to different categories (indoors and outdoors) that depict the coarse-to-fine and fine-to-coarse movies. Mean amplitude spectra of each categories. On each amplitude spectrum, the low spatial frequencies are close to the center, while the high spatial frequencies are in the periphery. The vertical orientations are represented on the x-axis while the horizontal orientations are represented on the y-axis.

Stimuli consisted of 40 black and white photographs (256-level grey-scales) of natural scenes classified into two distinct categories (20 indoor scenes and 20 outdoor scenes) with a visual angle of 24×18 degrees. Exemplar from the two categories (outdoor and indoor) were chosen in order to have similar amplitude spectrum to avoid their identification on the basis of this type of visual cue [21], but also to avoid contrast energy differences between categories that could interfere with the sequence of spatial frequency processing. In both categories, images have the same distribution of energy in spatial frequencies and dominant orientations (as shown by the mean amplitude spectrum of non-filtered natural scenes in each category; Figure 1). Stimuli were elaborated using the image processing toolbox on MATLAB (Mathworks Inc., Sherborn, MA, USA). We presented brief movies containing a succession of spatial frequency filtered scenes, going either from lower to higher frequency or vice versa. This allowed us to experimentally “decompose” the visual inputs in either CtF or FtC sequences. For each scene, we created two movies: one following a CtF sequence (see Video S1 and S3) and one following a FtC sequence (see Video S2 and S4). Each movie lasted 150 ms and was composed of the same scene filtered in 6 different frequency bands (presented 25 ms). Scenes were filtered using Gaussian band pass filters with different central frequencies equivalent to a visual angle of to 1, 2, 3, 4, 5, 6 cycles/degree, and a standard deviation of 1.67 cycles/degree (or 24, 48, 72, 96, 120, 144 cycles/image and a standard deviation of 40 cycles/image). The cut off frequencies at 67% of the height of each Gaussian were, therefore, [0 2.7]; [0.3 3.7]; [1.3 4.7]; [2.3 5.7]; [3.3 6.7]; [4.3 7.7] cycles/degree; (i.e. [0 64]; [8 88]; [32 112]; [56 136]; [80 160]; [104 184] cycles/image). Stimuli were displayed using E-prime software (E-prime Psychology Software Tools Inc., Pittsburgh, USA) on a computer monitor (17-inch, with a resolution of 1024×768 pixel size, 75 Hz) at a viewing distance of 73 cm. In order to respect the distance and the central position, participants' heads were supported by a chin rest.

### Procedure

The experiment consisted of 80 trials. Each image was perceived both in CtF and FtC sequences. In order to prevent any order effect, CtF and FtC sequences were randomized between participants. Each trial began with a central fixation point for 500 ms accompanied by a sound to focus attention, immediately followed by a movie lasting 150 ms, and a mask (white noise) for 300 ms. The quality of the central fixation was controlled by the experimenter. Participants had to make a categorical choice. They had to decide whether the scene was an indoor or an outdoor scene by pressing on the corresponding response buttons (aligned with the mid-sagittal plane of each participant) using the forefinger and the middle finger of their dominant hand. Half of the participants had to answer “indoor” with the forefinger and “outdoor” with the middle finger, while the second half of the participants had to answer ‘indoor” with the middle finger and “outdoor” with the forefinger. Reaction times, and response error rates, were recorded to the nearest millisecond (ms) following response. Before testing, each participant performed 8 training trials.

## Results

Two 2×2×2 variance analyses (ANOVA), with Sequence of spatial frequency processing (CtF and FtC) and Category (indoor and outdoor) as within-subject factors, and Age (young and aged participants) as between-subject factors were conducted on mean error rates (mER) and mean correct reaction times (mRT). It should be noted that the gender of participant did not interact with any of the interest factors (Sequence, Category and Age) and was not included in the analysis.

**Figure 2:**
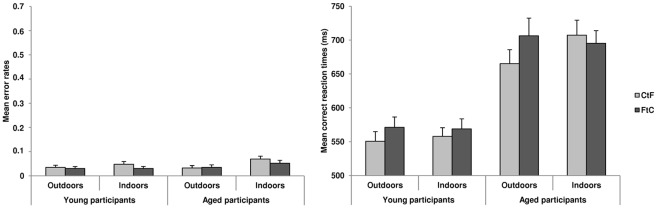
Mean error rates and mean correct reaction times in ms according to coarse-to-fine (CtF) and fine-to-coarse (FtC) sequences and scene categories (Outdoors and Indoors) for young and aged participants. Error bars correspond to standard errors.

The ANOVA conducted on mER (Figure 2) showed a main effect of neither Age (young participants: 4±5%; aged participants: 5±6%; F_1,44_ = 1.91, p = 0.17) nor Sequence of spatial frequency processing (CtF: 5±5%; FtC: 4±5%; F_1,44_ = 1.77, p = 0.19). However, we observed a main effect of categories (F_1,44_ = 6.46, p<0.05). Participants made more errors when categorizing indoor (5±5%) than outdoor scenes (3±5%). No interaction was observed between Sequence and Age (F_1,44_<1), Sequence and Category (F_1,44_ = 1.18, p = 0.28), and Sequence, Age and Category (F_1,44_<1).

The ANOVA conducted on mRT (Figure 2) showed that young participants categorized stimuli more quickly than aged participants (young participants: 562±68 ms; aged participants: 693±106 ms; F_1,44_ = 28.57, p<0.001). mRT were faster for CtF than FtC sequences (CtF: 620±109 ms; FtC: 635±112 ms; F_1,44_ = 11.71, p<0.01). The Sequence×Category interaction was significant (F_1,44_ = 8.84, p<0.01). Planned comparisons showed an advantage for CtF sequences in outdoor scenes irrespective of age (CtF-outdoor: 607±103 ms; FtC-outdoor: 638±122 ms; F_1,44_ = 19.32, p<0.001) but not for indoor scenes (CtF-indoor: 632±115 ms; FtC-indoor: 632±102 ms; F_1,44_<1). The Sequence×Age interaction was not significant (F_1,44_<1). Indeed, for young participants, mRT were faster for CtF than for FtC sequences (CtF: 554±65 ms; FtC: 571±70 ms; F_1,44_ = 6.33, p<0.05) and aged participants (CtF: 686±104 ms; FtC: 701±108 ms; F_1,44_ = 5.39, p<0.05). Interestingly, the Sequence×Category×Age interaction was significant (F_1,44_ = 4.32, p<0.05) due to a significant Sequence×Category interaction for aged participants only (young participants: F_1,44_<1; aged participants: F_1,44_ = 12.76, p<0.001). This result indicates an effect of categories on the sequence of spatial frequency in normal aging. Planned comparisons indicated that in aged participants, categorization of CtF-outdoor was significantly faster than FtC-outdoor sequence categorization (CtF-outdoors: 665±99 ms; FtC-outdoors: 706±125 ms; F_1,44_ = 17.17, p<0.001), whereas they categorized FtC-indoor faster than CtF-indoor sequences, even if this difference did not reach significance level (CtF-indoors: 707±107 ms; FtC-indoor: 695±90 ms; F_1,44_ = 1.54, p = 0.22).

## Discussion

The main aim of this behavioral study was to provide supplementary arguments in favor of the predominantly coarse-to-fine visual sequence processing supported by recent visual models and data [7], [16], [18], [42], [43], using for the first time movies which experimentally “mimic” the sequential processing of spatial frequencies postulated by these models.

The results obtained on reaction times demonstrated that participants categorized CtF sequences more quickly than reverse FtC sequences. These data are consistent with behavioral studies showing a global precedence using hierarchical stimuli [23], or a temporal precedence of LSF on HSF processing using static scenes [18]. However, in the Schyns and Oliva's study [18], participants viewed a filtered scene (in LSF or HSF) or a hybrid image followed by a non-filtered scene, and had to decide whether both scenes belonged to the same category. Thus, spatial frequencies were not presented sequentially as postulated in the CtF hypothesis. To test the CtF processing scheme, and to identify its neural substrates, Peyrin et al. [16], [17] presented sequences of two spatial frequency filtered scenes in rapid succession (LSF followed by HSF or vice versa) during fMRI and ERPs. Participants had to judge whether the two successive scenes belonged to the same category. This procedure allowed researchers to experimentally “mimic” and to impose different sequence of spatial frequency processing, and to assess neural responses to LSF and HSF presented in different order. However, the two scenes in each sequence were displayed for a long time (100 ms) with an inter-image interval of sufficient length (400 ms) to allow complete processing of the first image and to avoid an overlap of brain responses to the two images during ERP recordings. This procedure was, therefore, obviously not physiological. In the present study, we presented sequences of six spatial frequency filtered scenes in rapid succession, going from lower to higher frequencies, or vice versa, within a movie for better simulation of the dynamic of visual processing. The first milliseconds of the CtF movies provided LSF information and the coarse structure of the scene. This information was conveyed by the fast magnocellular pathways, allowing an initial perceptual categorization. The last milliseconds of the CtF movies provided HSF information and the fine structure of the scene. This information was conveyed by the slow parvocellular pathways, allowing refinement of the initial categorical choice which was based only on LSF information. In FtC movies, HSF information displayed at the beginning of the movie did not provide enough relevant information to allow rapid categorization.

However, we observed a significant Sequence×Category×Age interaction due to an effect of categories on sequence processing for aged participants only. This result suggests that the strategy of spatial frequency processing (either CtF or FtC) used by aged participants was modulated by the category involved. Indeed, young participants categorized CtF movies more quickly than FtC movies irrespective of category, while aged participants were faster to categorize CtF movies of outdoor scenes, but had a tendency to categorize FtC movies of indoor scenes more quickly than their CtF counterparts. This interaction could be interpreted in terms of flexibility of perceptually-driven spatial frequency processing. The authors Mermillod et al. [22] observed, for example, that the categorization of close natural scenes composed of many perceptual elements, such as indoor scenes, was based on HSF, whereas the categorization of open natural scenes without fine perceptual elements, such as city scenes, was based on LSF. Thus, the openness of a scene could guide the spatial frequencies used in categorization. In our study, although the categories used were equivalent in terms of their various physical properties, such as the distribution of energy in dominant orientations in the Fourier domain, differences between indoor and outdoor scenes can be pinpointed in the spatial domain, particularly in the visual organization of the elements which make up a scene. In fact, the visual organization of the different elements in outdoor scenes (man-made outdoors and street views) is very similar. By averaging outdoor scenes, coarse information such as the ground, the sky and the direction of natural light remains salient and could guide categorization. Such spatial regularities are not present in indoor scenes. Indeed, the photographs of the indoor scenes included many local elements (e.g., table, sofa, stair-case) in different spatial organizations with the potential to alter recognition of the scenes regardless of their spatial frequencies. In fact, our results showed that participants made significantly more errors in the categorization of indoor compared to outdoor scenes. Thus, in the spatial domain, LSF in outdoor movies provide the relevant information, allowing an efficient rapid categorization with respect to the invariant global structure (i.e., ground and sky) in accordance with a predominant CtF processing sequence. For indoor movies, LSF was unable to provide sufficient information to allow efficient rapid categorization, and HSF may be preferentially used to process local elements. Thus, the categorization of scenes could well be based on additional cues (e.g., spatial organization of the elements composing the scene) that might interfere with the spatial frequency processing.

Based on this assumption, we hypothesized that the nature of visual information extracted from the scene for a rapid categorization varies with age. For young participants, categorization seems to be based mainly on the overall spatial frequency content, and a default CtF strategy is used regardless of the category. This is consistent with results obtained by Schyns and Oliva [18], showing an overall CtF strategy among young participants irrespective of the category used. On the contrary, aged participants seem to use additional information to categorize scenes, such as the spatial properties of the image (blobs that depict spatial invariants for outdoors, and lines that define local elements for indoors). Aged participants are significantly slower than young participants (reaction times were longer by 100 ms), and this is consistent with an additional visual process. In this way, they may categorize CtF movies of outdoors in which blobs are available at the beginning of the movie more quickly in LSF, while they might categorize FtC movies of indoors in which lines are available at the beginning more quickly in HSF. In short, with increasing age, the nature of the visual information extracted during the first milliseconds of the movies may vary with the intrinsic properties of the categories used, and this emphasizes the importance of considering different semantic categories of scenes when investigating visual sequences of categorization. In order to explore this assumption, in the future it will be necessary to test different categories characterized by different spatial organizations. We could, for instance, contrast forest scenes containing many details (e.g., trees, leaves) with empty field scenes, and expect categorization sequences based on LSF for fields, and based on HSF for forests.

These data are of importance when considering quality of life in elderly people. They also provide interesting perspectives for the investigation of locomotion of patients in indoor and outdoor environments. Interestingly, studies on home environment in the elderly have expanded significantly in recent scientific literature. Indeed, the majority of seniors prefers to “age in place” and spends most of its time at home [44]. Quality of life at home became a public health issue, and the study of home environment appears as an interesting framework for understanding individual functioning [45], [46]. In particular, studies have shown that improving the environment had a beneficial effect on fall risk [47], [48], [49], [50]. Indeed elderly people seem to apprehend indoor and outdoor environments differently from younger people. In outdoor environments, elderly people may detect invariants mainly conveyed by LSF information, and this could help them to navigate and move within these environments. However, they may focus on details (e.g., objects) to be located in indoor environments. These data on the visual sequences of the elderly are important to consider in regard to pathological aging. For example, age-related macular degeneration disease (AMD) affects mainly the central vision of people over the age of 50 [51], [52], [53], [54]. The investigation of categorization sequences guided by spatial frequencies is even more important since we showed recently that elderly people with AMD have difficulty in processing HSF information, mostly in indoor scenes [55].

### Conclusion

The current study provides new arguments for predominantly CtF categorization of natural scenes, using for the first time dynamic sequences intended to simulate the time-course of spatial frequency processing within the visual system. Moreover, the sequence of spatial frequency processing appears to be perceptually driven by the nature of the stimuli with increasing age. Aged participants use coarse information to categorize outdoor scenes but tend to focus on detailed information to categorize indoor scenes. Our results suggest that the visual sequence of spatial frequency processing may well be constrained by the spatial organization of elements and their regularities within the different categories of natural scenes in aged participants.

## Supporting Information

Video S1 Example of stimuli “CtF Outdoor”.
(AVI)

Video S2 Example of stimuli “FtC Outdoor”.
(AVI)

Video S3 Example of stimuli “CtF Indoor”.
(AVI)

Video S4 Example of stimuli “FtC Indoor”.
(AVI)
